# Feasibility of Reconstructing Source Functional Connectivity with Low-Density EEG

**DOI:** 10.1007/s10548-021-00866-w

**Published:** 2021-08-20

**Authors:** Dung A. Nguyen-Danse, Shobana Singaravelu, Léa A. S. Chauvigné, Anaïs Mottaz, Leslie Allaman, Adrian G. Guggisberg

**Affiliations:** Imaging-Assisted Neurorehabilitation Lab, Department of Clinical Neurophysiology, University of Geneva, Av. de Beau-Séjour 26, 1211 Geneva, Switzerland

**Keywords:** Functional connectivity, Low–density, Electroencephalography, Alpha oscillations, Neurofeedback

## Abstract

**Objectives:**

Functional connectivity (FC) is increasingly used as target for neuromodulation and enhancement of performance. A reliable assessment of FC with electroencephalography (EEG) currently requires a laboratory environment with high-density montages and a long preparation time. This study investigated the feasibility of reconstructing source FC with a low-density EEG montage towards a usage in real life applications.

**Methods:**

Source FC was reconstructed with inverse solutions and quantified as node degree of absolute imaginary coherence in alpha frequencies. We used simulated coherent point sources as well as two real datasets to investigate the impact of electrode density (19 vs. 128 electrodes) and usage of template vs. individual MRI-based head models on localization accuracy. In addition, we checked whether low-density EEG is able to capture inter-individual variations in coherence strength.

**Results:**

In numerical simulations as well as real data, a reduction of the number of electrodes led to less reliable reconstructions of coherent sources and of coupling strength. Yet, when comparing different approaches to reconstructing FC from 19 electrodes, source FC obtained with beamformers outperformed sensor FC, FC computed after independent component analysis, and source FC obtained with sLORETA. In particular, only source FC based on beamformers was able to capture neural correlates of motor behavior.

**Conclusion:**

Reconstructions of FC from low-density EEG is challenging, but may be feasible when using source reconstructions with beamformers.

## Introduction

Interregional neural communication is thought to be accompanied by a synchronization of oscillations between different brain regions (Aertsen et al. 1989; Varela et al. 2001). This interregional synchronization can be quantified with the concept of “functional connectivity” (FC) which is a measure of statistical dependency between activity in different brain regions and is therefore considered to be an index of functional interaction (Nunez et al. 1997; Varela et al. 2001; Nolte et al. 2004). FC magnitude computed from fMRI was found to correlate with performance, e.g., with reading competency, executive function, and episodic memory capacity (Wang et al. 2010; Koyama et al. 2011; Reineberg et al. 2015). fMRI-FC was also associated with motor performance in a sit-to-stand-to-sit task in low back pain patients (Pijnenburg et al. 2015). In electroencephalography (EEG), neural assemblies spontaneously produce prominent oscillations at a frequency of about 8 to 12 cycles per second even when the recorded subjects are at rest (Hindriks et al. 2014), which is also the preferred frequency for interregional communication (Chapeton et al. 2019). Alpha-band FC between a brain area and the rest of the brain (i.e., the node degree of a brain area) correlates with behavioral performance in healthy participants (Guggisberg et al. 2015), and with neurological deficits in patients (Dubovik et al. 2012). For instance, the more spontaneous alpha activity in Broca’s area is coherent with the rest of the brain, the better subjects are able to produce words. Recent findings have demonstrated that spontaneous FC in the alpha band enables particularly high performance and is thus a better predictor of performance than classical task-induced activations (Allaman et al. 2020).

Thus, FC is increasingly used as marker to probe for novel disease biomarkers (Fox and Greicius 2010) or predictors of outcome (Westlake et al. 2012; Nicolo et al. 2015). Furthermore, it has become a target for new treatment approaches aiming at enhancing performance or reducing neurological deficits. Patients were able to improve their motor function when asked to enhance the FC in their motor cortex; which was not the case when a non-related brain area was trained (Mottaz et al. 2018). However, the setup of a neurofeedback as performed in a laboratory is time-consuming and tiring for the patients. Not only it requires individual head models based on magnetic resonance imaging (MRI), but also high-density electroencephalography (hd-EEG). Such installation is hardly feasible in routine clinical practice or at a larger scale.

For end-users and patients to use EEG in clinical settings, the montage must be user-friendly and easily set up. One solution is to reduce the number of channels, which reduces the setup time and makes the entire EEG installation more mobile. Motor-disabled patients have successfully used low-density EEG for brain-computer interface (BCI) tasks (Leeb et al. 2013). In clinical practice, low-density EEG is already part of standard practice. For diagnosis purposes, 9 to 21 electrodes are used in epileptology (Koutroumanidis et al. 2017; Rossetti et al. 2012; Wilmshurst et al. 2015).

However, reducing the number of electrodes is more delicate when the aim is to reconstruct FC. The electromagnetic potential spreads throughout the brain because of volume conduction and is thus received to some degree by all sensors. This leads to a mix of signals from multiple brain areas at each electrode, which makes the targeting of a given brain lobe of interest difficult. Furthermore, volume conduction induces a massive overestimation and distortion of FC (Srinivasan 2007; Schoffelen and Gross 2009). The computation of inverse solutions allows to partially revert volume conduction and obtain the signal at the source. This gives access to signals at regions of interest and reduces volume conduction issues in the computation of FC. Yet, the usage of inverse solutions usually requires a good spatial sampling and thus hd-EEG recordings (Michel et al. 2004).

This study aimed to evaluate the feasibility of using a low-density EEG (with 19 channels) for reconstructing FC markers of performance and disability in the example of motor performance. Reducing the number of channels would reduce the time spent on the installation, de-installation, maintenance, and care of the participant and the whole equipment. If feasible, we could then make FC markers and treatment targets accessible to clinical practice. To this end, we will need to satisfy two main requirements. First, we need to be able to capture variance in FC magnitude, such that periods or subjects having lower neural coupling show proportionally lower values of reconstructed FC. Second, we need to have some fidelity in localization accuracy such that neural coupling at a target brain area is correctly captured.

We compared different technical approaches to the problem. Mixed signals captured by average and Laplacian re-referenced (McFarland et al. 1997) electrodes were used to compute sensor FC. To obtain unmixed signals we used inverse solutions (Pascual-Marqui 2002; Sekihara et al. 2004) and independent component analyses (ICA), which allowed computing FC between sources or independent components, respectively. Each of these approaches was then evaluated with regards to localization error and with regards to the correlation of reconstructed FC magnitude with behavior. This was first done with numerical simulations and then tested in different real datasets.

## Materials and Methods

### Datasets

The study comprised 3 datasets.

- *Dataset 1*: 70 healthy subjects (45 women, 37.9 ± 17.6 years old) underwent resting-state EEG recording with a 128 channel Biosemi ActiveTwo EEG-system (Biosemi B.V., Amsterdam, Netherlands).
- *Dataset 2*: 20 healthy subjects (13 women; 28.7 ± 5.6 years old) underwent resting-state EEG recording with a 128 channel Biosemi ActiveTwo EEG-system and behavioral assessment of motor performance with a finger-tapping task. All had a normal or corrected-to-normal vision and no history of neurological or psychiatric disorders and were paid for their participation.
- *Dataset 3*: 20 healthy subjects (17 women, 25.5 ± 5.4 years old) underwent resting-state EEG recording with a 20 channel Enobio system with dry-gel electrodes (neuroelectrics, Barcelona) and behavioral assessment of motor performance with a finger-tapping task. All had a normal or corrected-to-normal vision and no history of neurological or psychiatric disorders and were paid for their participation.

All procedures were approved by the ethical committee of the canton of Geneva and performed according to the declaration of Helsinki. All participants gave written informed consent after receiving an explanation on the nature of the experiments.

### Behavioral Assessments

Participants of dataset 2 and 3 performed a sequential finger-tapping task (FTT) (Zhang et al. 2012) immediately after the resting-state EEG recording. The task was designed using E-Prime 2.0 software (Psychology Software Tools, Pittsburgh, PA). Participants were instructed to repeat a given five-item sequence with their left hand (little finger to index) on four horizontally arranged buttons numbered left to right on a Chronos box (Psychology Software Tools, Pittsburgh, PA; https://pstnet.com/products/chronos/). The same sequence was used throughout the whole experiment (1-4-2-3-1). It was continuously presented to participants while they had to perform it. They had to repeat the sequence as fast and accurately as possible during two blocks of 30 s. No feedback was given. Motor performance was quantified as the average number of correct motor sequences per minute.

### Recordings

EEG was recorded with a 128-channel Biosemi ActiveTwo EEG-system using active gel electrodes (Biosemi B.V., Amsterdam, Netherlands) at a sampling rate of 512 Hz in datasets 1 and 2, or with a 20-channel Enobio system using dry-gel electrodes (neuroelectrics, Barcelona) at 500 Hz in dataset 4 in an awake, resting condition during which subjects kept their eyes closed. Artifacts and data segments with signs of drowsiness were excluded by visual inspection of the data. Electrodes containing artifacts persistent across multiple epochs were excluded. These electrodes were interpolated from neighboring electrodes using a 3D spline interpolation (< 5% interpolated electrodes) (Perrin et al. 1987) for analyses of sensor FC and for source reconstructions with standardized low-resolution electromagnetic tomography (sLORETA), but not for source reconstruction with beamformers.

The 19 electrodes followed the positions of the international 10–20 system. In datasets 1 and 2, they were selected from the full 128 montage. For comparison, sensor FC was also computed from sensor data with an average reference and a small Laplacian reference of each electrode.

The EEG was segmented into 300 non-overlapping, artifact-free epochs of 1 s duration and bandpass-filtered between 1 and 20 Hz.

**Fig. 1:**
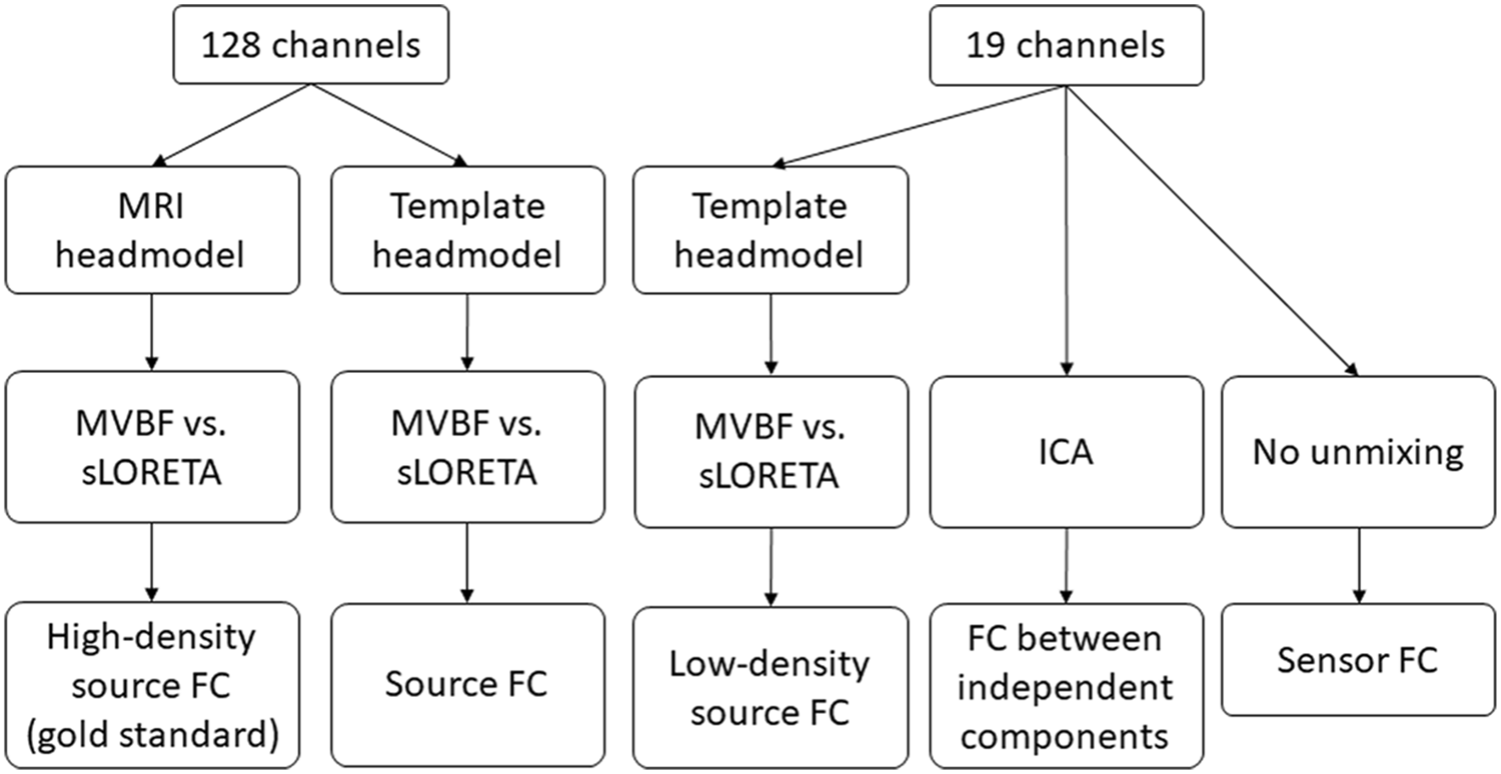
Schematic overview of analysis setups. High-density montages with 128 electrodes were compared to low-density subsets or systems with 19 electrodes, according to the international 10–20 standards. Unmixing of electrode signals was performed with either source localization or independent component analysis (ICA) and compared to the performance obtained without unmixing. *MVBF* minimum variance beamformer, *sLORETA* standardized low-resolution electromagnetic tomography, *ICA* independent component analysis, *FC* functional connectivity

Figure 1 gives a schematic overview of the different analysis steps that were taken in order to reconstruct FC in real data from low-density recordings, as compared to the high-density montages as gold standard.

### Source Localization

For individual MRI-based head models, the MRI protocol contained a high-resolution T1-weighted, 3-D spoiled gradient-recalled echo in a steady state sequence covering the whole skull (192 coronal slices, 1.1 mm thickness, TR = 2500 ms, TE = 3 ms, flip angle = 8°).

Each subject’s brain was segmented into scalp, skull, grey and white matter with *NUTMEG* (http://nutmeg.berkeley.edu) (Dalal et al. 2011) and the toolbox *MARS* (https://www.parralab.org/mars/) (Huang and Parra 2015).

We computed the lead-potential with 10 mm grid spacing (~ 1200 solution points) using a boundary element head model (BEM). The BEM was created with the *Helsinki BEM library* (http://peili.hut.fi/BEM/) (Stenroos et al. 2007) and the *NUTEEG* plugin of *NUTMEG*, based on the individual T1 MRI of each subject as well as based on the Montreal Neurological Institute template brain.

Source FC was calculated in *Matlab* (The MathWorks Inc., Natick, USA) with *NUTMEG* (http://nutmeg.berkeley.edu) (Dalal et al. 2011) and its *functional connectivity mapping* (*FCM*)* toolbox* (Guggisberg et al. 2011).

Most previous studies reporting correlations between alpha-band FC and behavior, and previous trials using neurofeedback to train alpha-band FC were based on source reconstructions of hd-EEG arrays with beamformers (Dubovik et al. 2012; Guggisberg et al. 2015; Mottaz et al. 2015, 2018). Thus, we used the same approach here as a reference for comparison with other approaches. A *scalar minimum variance beamformer* (MVBF) was used to project preprocessed hd-EEG data to source space (Sekihara et al. 2004).

The MVBF uses the temporal covariance of the EEG data (in addition to the sensor geometry) to create a custom spatial filter depending on the signal characteristics. This enables more precise and focal source localization (Sekihara et al. 2005). However, beamformers are sensitive to the accuracy of the head model; measured data that is inconsistent with the head model is liable to be rejected as noise (Steinsträter et al. 2010). We thus compared beamformers to standardized low-resolution electromagnetic tomography (*sLORETA*) (Pascual-Marqui 2002) as a widely used inverse solution that does not have these limitations of beamformers. On the downside, it enables less focal reconstructions (Sekihara et al. 2005) and thus is likely to induce more spatial leakage of the reconstructed sources.

### Dipole Orientation

For the localization of FC in the brain, we require an estimation of neural network oscillations at each solution point. Vector weights obtained from the inverse solution allow only reconstructing squared power values; the reconstruction of neural *oscillations* requires a scalar weight matrix. In order to scalarize the lead-potential as input to scalar weights computation, we need to determine the dipole orientation at each grid location. We computed the optimal dipole orientation at each solution point as the orientation yielding maximum output signal-to-noise ratio (SNR). For the MVBF, the optimum orientation at each solution point *v* is given by (Sekihara et al. 2004):

1 $${{\varvec{\eta}}}_{v}={{\varvec{v}}}_{min}\left\{{\left[{{\varvec{L}}}_{v}^{T}{{\varvec{R}}}^{-1}{{\varvec{L}}}_{v}\right]}^{-1}\left[{{\varvec{L}}}_{v}^{T}{{\varvec{R}}}^{-2}{{\varvec{L}}}_{v}\right]\right\}$$

where ***v***_*min*_ is the eigenvector corresponding to the minimum eigenvalue of the matrix in {}, ***L*** is the vector lead-potential, **R** the sensor covariance matrix, and superscript *T* denotes the matrix transpose.

For sLORETA, it is obtained as (Pascual-Marqui et al. 2009):

2 $${{\varvec{\eta}}}_{v}={{\varvec{v}}}_{max}\left\{{{\varvec{L}}}_{v}^{T}{{\varvec{G}}}^{-1}{{\varvec{R}}{\varvec{G}}}^{-1}{{\varvec{L}}}_{v} , {{\varvec{L}}}_{v}^{T}{{\varvec{G}}}^{-1}{{\varvec{L}}}_{v}\right\}$$

where ***v***_*max*_ is the eigenvector corresponding to the maximum generalized eigenvalue, and **G** the gram matrix defined as ***LL***^*T*^.

The scalar lead-potential was then calculated as:

3 $${{\varvec{l}}}_{v,\eta }={{\varvec{l}}}_{v}{{\varvec{\eta}}}_{v}$$

### Independent Component Analysis (ICA)

EEG electrodes carry information from multiple brain sources that are mixed together. One approach to unmix these signals is independent component analysis (Hyvarinen 1999; Reineberg et al. 2015). Average referenced sensor time series with 19 channels of all subjects were normalized to common amplitude and variance using z-scores, concatenated in the time dimension, and subjected to a *FastICA* algorithm (Hyvarinen 1999) with the *FastICA* package for *MATLAB* (https://research.ics.aalto.fi/ica/fastica/). Default parameters were used. This led to 19 independent components. The unmixing matrix obtained from all subjects was then applied to the sensor data of each subject.

### Functional Connectivity (FC)

We used the absolute imaginary component of coherence (IC) as an index of FC (Nolte et al. 2004; Sekihara et al. 2011). One FC value was obtained from all 300 epochs of data. For sensor FC, it was computed directly between the preprocessed and filtered EEG sensor data. For ICA data, IC was computed between independent components. For source FC, we used the source time series estimated with inverse solutions. From this, we calculated the weighted node degree (WND) for each voxel, component, or electrode as the mean of its coherence with all other voxels/components/electrodes (Newman 2004). The WND can been as index of the overall importance of a brain area in the network.

FC values can be influenced by the signal-to-noise ratio of the EEG. To minimize this potential confound, we normalized WND values at each voxel by subtracting the mean WND across all voxels and then dividing by the standard deviation of all voxels, thus obtaining z-scores (Dubovik et al. 2012; Mottaz et al. 2015).

In case of individual head models, normalized WND values were spatially normalized to canonical Montreal Neurological Institute space with functions from the Matlab toolbox SPM8 (https://www.fil.ion.ucl.ac.uk/spm/software/spm8/).

### Regions, Independent Components, and Electrodes of Interest

For correlations between source FC and visuo-motor behavior, we defined the right (i.e. contralateral to the moved hand) Precentral gyrus as region of interest (ROI) using the automated anatomical labeling (AAL) atlas (Tzourio-Mazoyer et al. 2002). ROI values were obtained as the average WND across its containing voxels. For sensor FC, electrode C4 was analyzed. For ICA processed data, we defined an independent component of interest. For this, we correlated source time series obtained with the full EEG array, individual head models, and MVBF with the time series of each independent component. The independent component whose time series correlated positively with source time series at the right sensorimotor cortex was used for further analysis.

### Statistical Analyses

To investigate the fidelity of source reconstructions with template head models and with low-density EEG arrays, we correlated normalized WND values obtained with individual MRI-based head models and 128 channel data (gold standard) to normalized WND values obtained with template head models using 128 or 19 electrodes. Pearson correlation coefficients were computed over all 82 cortical ROIs of the AAL atlas for each of the 70 subjects of dataset 1, i.e., the normalized WND values of the gold standard at all ROIs were correlated with the values of the test setup at all ROIs. Fisher-Z transformed correlation coefficients of all subjects were then fed to a one-way ANOVA with the analysis setup as a dependent factor. Pairwise post-hoc comparisons were done with the Tukey–Kramer HSD correction.

For comparison between sensor and source FC, we matched each of the 19 electrodes of the international 10–20 system to the ROI that had the shortest Euclidean distance from the electrode’s position. Source normalized WND obtained from 19 electrodes at these ROIs as well as sensor normalized WND obtained from 19 average-referenced or Laplacian-referenced electrodes was then correlated to the normalized WND values obtained with individual MRI-based head models and 128 channel data.

We also correlated source FC at the right precentral gyrus obtained with template head models and 19 electrodes to the source FC obtained with the gold standard, since this was our ROI for motor behavior. This time, the correlation was done over subjects.

Next, we investigated the ability of low-density EEG arrays to capture associations with behavioral performance. Pearson correlation coefficients were computed for associations between alpha-band FC and FTT performance in subjects of datasets 2 and 3. This was done for the precentral ROI, C4 electrode, and the independent component of interest. The correlation obtained with the gold standard (individual MRI-based head models, 128 channel data, MVBF) was juxtaposed to the correlation coefficients obtained with 19 channels using either source localization, sensor FC, or FC between independent components.

To check the feasibility of obtaining FC using even more convenient dry-gel electrodes, we then used data from patients in dataset 3 to correlate source FC with FTT performance.

### Numerical Simulations

For 38 randomly selected subjects of dataset 1, we simulated 3 cortical point sources with a 10 Hz sinusoidal rhythm. The main source of interest was placed to the center of the right primary motor cortex. Two additional sources with 10 Hz oscillation were defined at the left primary motor cortex and the right premotor area. They had a radial phase lag of π/2 (= 25 ms) or − π/2, respectively, relative to the first point source. This phase difference leads to maximal values in the imaginary part of coherence, while the lag of π between sources 2 and 3 produce 0 imaginary coherence. The dipole orientations were fixed to point in a random orientation at each location. In addition, to test whether our settings are able to capture variance in coupling strength across subjects, we additionally simulated variance in coupling strength between point source 1 and point sources 2/3. This was achieved by simulating alpha activity in only 60% of the 300 epochs. The number of epochs with alpha activity at both source 1 and sources 2/3 at the same time (i.e., with coherent alpha activity) was then varied across subjects between a minimum of 57% and maximum of 100% of alpha epochs. Thus, the remaining epochs contained alpha activity either only at source 1 or only at sources 2/3. The cortical sources were then projected to the EEG sensors by using a scalar lead-potential calculated with a BEM head model based on individual MRIs. Four different levels of Gaussian random noise were added to the sensors (SNRs of 1, 2, 3, or 5). A total of 300 epochs of 1 s were created in this way to obtain the same data size as in real data. The simulated sensor data was then processed as the real data: it was bandpass filtered between 1 and 20 Hz, and projected back to all gray matter grid locations through a spatial filter matrix calculated with the MVBF and sLORETA inverse solutions described above. The WND of IC was computed at all cortical grid locations for the alpha frequency band as in the real data.

The Euclidean distance between the WND peak and the coordinate of source 1 was then calculated to determine the localization error. Errors were subjected to a three-way ANOVA with the setup (head model and number of channels), inverse solution, and SNR as dependent factors.

The coupling strength that was simulated was correlated with the magnitude of WND. Correlation coefficients were tested for difference between setups using Meng’s test for correlated correlations (Meng et al. 1992).

## Results

### Numerical Simulations

**Fig. 2:**
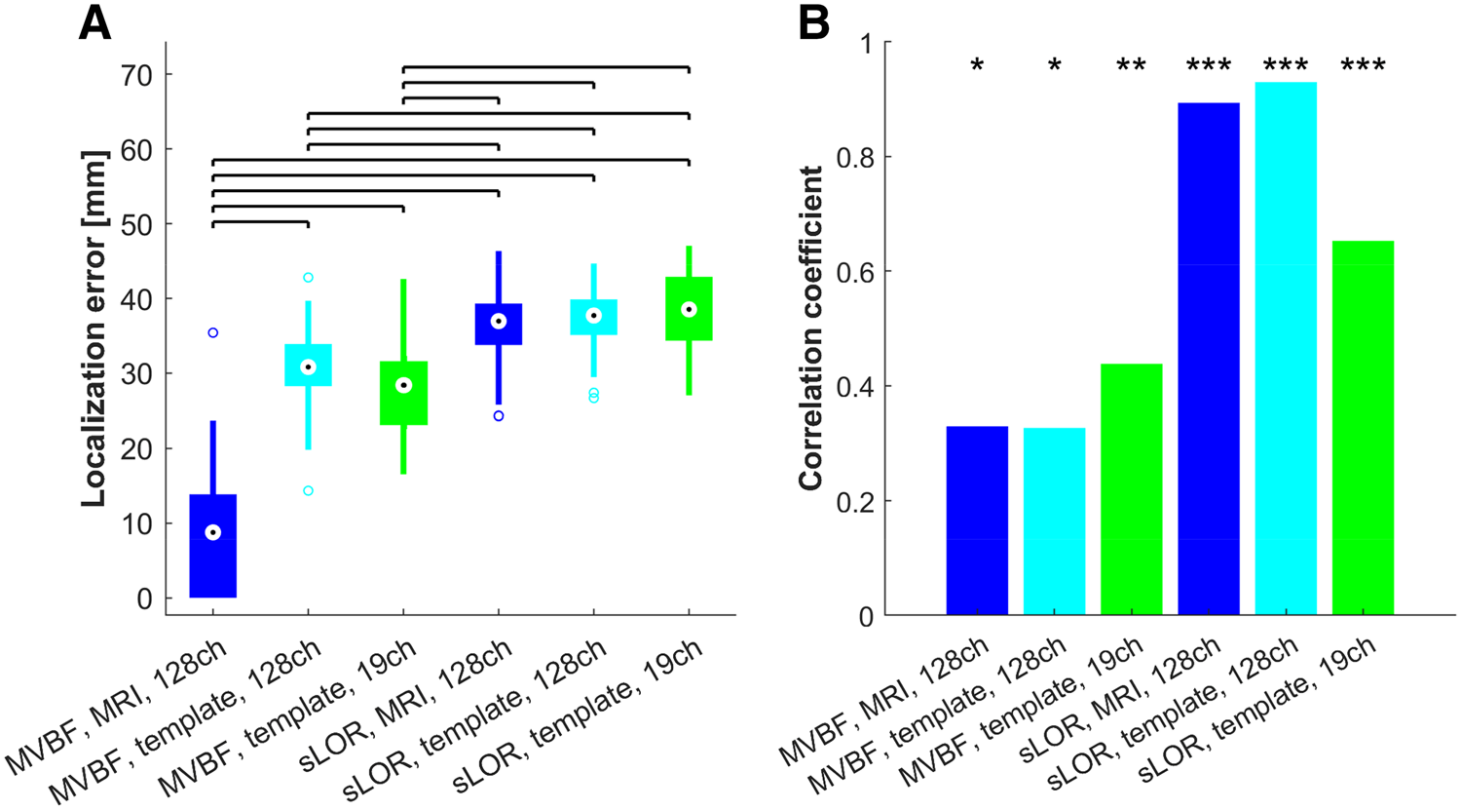
Results from simulations of 3 coherent point sources. **A** MVBF enabled better localization accuracy than sLORETA (sLOR), but was vulnerable to the usage of template head models and low-density montages. When using sLORETA, the accuracy remained stable when using only 19 electrodes. Horizontal bars indicate significant differences between setups (p < 0.05, Tukey Kramer HSD). **B** The magnitude of functional connectivity (FC) computed with all setups correlated with the simulated variations in coupling strength, thus confirming their ability to capture these variations. FC computed after source localization with sLORETA covaried better with the simulated coupling strength than FC obtained from MVBF, but showed vulnerability to usage of low-density montages

In simulations, we observed that the localization accuracy was significantly better when using MVBF than sLORETA at all SNRs (F_1,911_ = 351, p < 0.0001), in accordance with previous findings from high-density settings (Guggisberg et al. 2011). Furthermore, localization error was significantly influenced by the montage and head model (F_2,911_ = 85, p < 0.0001) with a rapid drop in localization accuracy when using a template head model and low density montages in MVBF. When using sLORETA, the accuracy was low already with MRI head models and 128 channels, but remained stable when using 19 electrodes and a template head model (Fig. 2A). The SNR of simulations did not significantly influence localization accuracy (p = 0.36).

All setups were able to capture the simulated variation in coupling strength, as indicated by a significant correlation between the simulated coupling and the computed FC (Fig. 2B). There was significant variation of the correlation coefficient between setups (χ^2^ = 64, p < 0.0001), but the correlation coefficient obtained with sLORETA and 19 electrodes did not significantly differ from the one obtained with MVBF and 19 electrodes (z = − 1.4, p = 0.92).

### Real Data

**Fig. 3:**
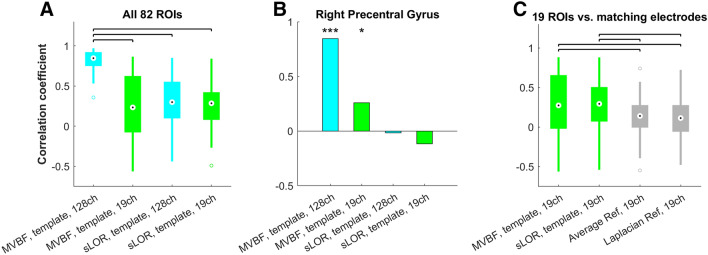
Fidelity of reproduction of source FC in real data. FC reconstructions obtained with 128 electrodes, individual MRI head models, and MVBF were used as gold standard and correlated to source FC obtained with other setups. **A** Box plot of correlation coefficients obtained in each subject across at all 82 cortical ROIs of the atlas. A drop in the average correlation coefficient occurred when using 19 electrodes or sLORETA (sLOR). **B** The same drop in reconstruction fidelity could be observed when focusing on the right Precentral gyrus. Bars indicate the correlation coefficient across subjects. **C** Using inverse solutions with 19 electrodes allowed better reliability of reconstructing FC than using sensor FC without source localization, no matter the reference. Box plots indicate the correlation coefficents obtained in each subject across electrodes for sensor FC (grey bars) or across the 19 cortical ROIs closest to each electrode for source FC (green bars)

When using MVBF to compute source FC, the usage of template head models led to only minor changes in the reconstructed FC across 82 cortical ROIs as indicated by high correlations to FC values obtained with individual MRI-based head models in individual subjects (r = 0.85 ± 0.03), see Fig. 3A. Conversely, MVBF was vulnerable to the usage of low density montages as indicated by lower correlations to the gold standard (r = 0.30 ± 0.38). Source FC obtained with sLORETA differed to those obtained with MVBF, even when using 128 electrodes and individual headmodels. Pearson correlations of ROI WND values in each subject revealed relatively low correlation coefficients on average (r = 0.35 ± 0.33). On the other hand, sLORETA was robust to reduction of the number of electrodes. Source FC obtained with sLORETA and 19 channels was similar to source FC obtained with an individual MRI-based headmodel and sLORETA (r = 0.73 ± 0.09), while the correlation with the MVBF gold standard (r = 0.30 ± 0.31) was similar as when obtained with 128 electrodes.

When focusing on the right Precentral ROI which was of primary interest here in the search for FC correlates of motor performance, we also observed a drop of FC reconstruction fidelity when using MVBF with low-density montages, while sLORETA had a generally poor performance (Fig. 2B).

Sensor WND values correlated poorly with the source WND values at the 19 ROIs that were closest to the positions of the electrodes. Using source reconstruction with MVBF or sLORETA based on the same 19 electrodes yields significantly better fidelity of FC (Fig. 3C).

**Fig. 4:**
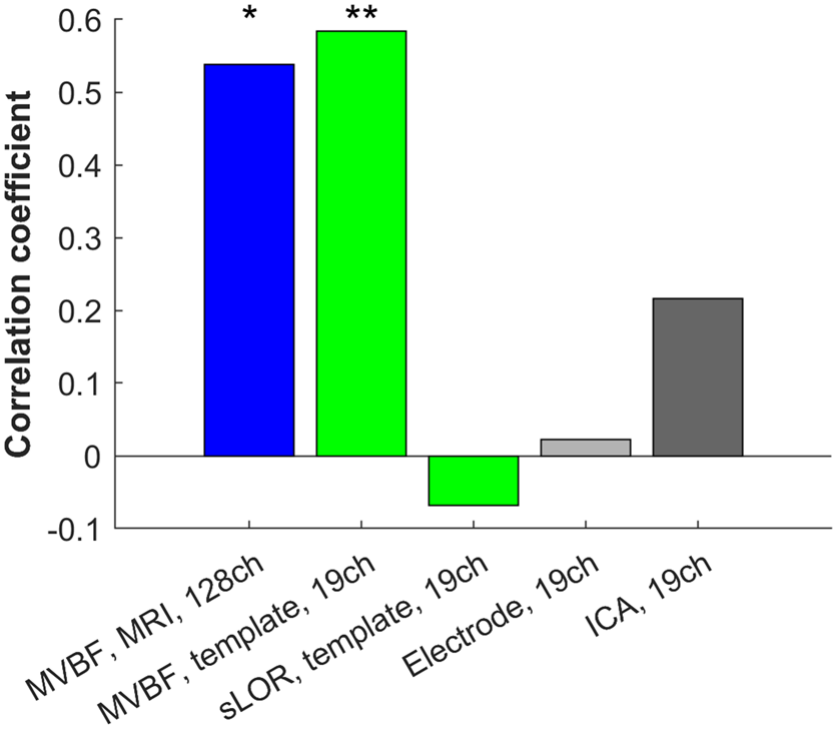
Correlation between FC and motor performance as quantified by the number of completed sequences per minute in a finger-tapping task (FTT) by 20 healthy subjects. Source FC derived after source localization with MVBF revealed significant FC correlates of motor behavior, even when using only 19 electrodes. This was not the case for source FC obtained from sLORETA (sLOR), sensor FC, or FC obtained after ICA

**Fig. 5:**
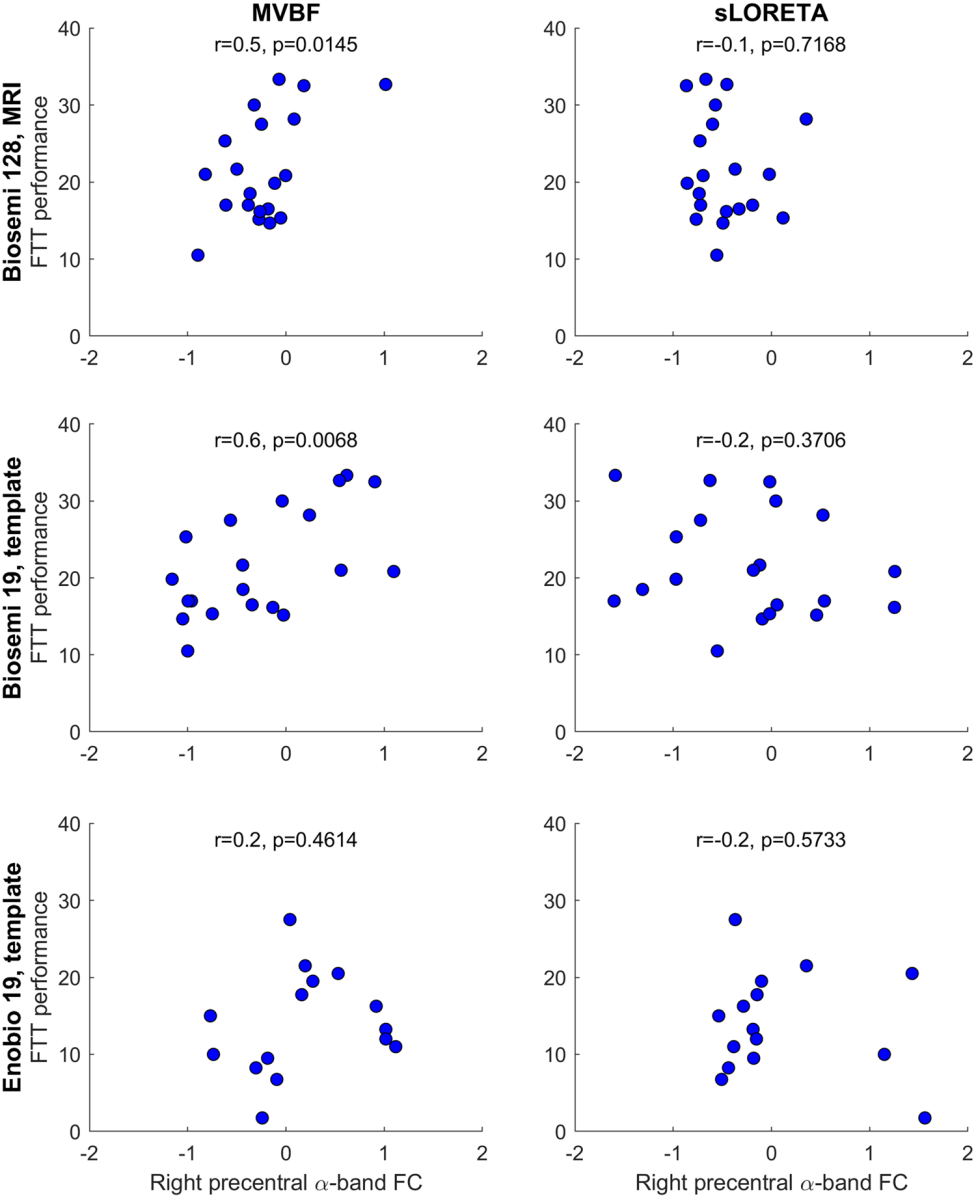
FC correlates of motor performance. Source FC reconstructed with MVBF from data recorded with active gel electrodes correlated with FTT performance, even when using template head models and 19 electrodes. Conversely, source FC computed with sLORETA or from 19 dry-gel electrodes was unable to capture correlates of motor performance

Next, we investigated the ability of low-density EEG to capture FC correlates of behavioral performance by computing the correlation between the reconstructed WND and motor performance in healthy subjects of dataset 2. Source WND reconstructed from 128 channel data with individual MRI-based head models and MVBF showed high correlations with motor performance at the precentral gyrus, as expected (Figs. 4 and 5). Using a subset of only 19 electrodes and a template head model did not reduce the ability to capture FC correlates of motor performance when performing source localization with MVBF. Conversely, this correlation was lost when doing source reconstruction with sLORETA. Similarly, sensor FC obtained without unmixing or FC computed between ICA were unable to reveal significant correlates of motor performance.

Finally, we intended to reproduce the findings of dataset 2 in independent dataset 3, this time using a low-density EEG system with dry-gel electrodes which are more convenient in setup than regular gel electrodes. Data from 5 subjects had to be excluded due to abundant artifacts and only data from the remaining 15 subjects were further analyzed. Figure 5 demonstrates that the correlations obtained in dataset 2 were not present in dataset 3, no matter the inverse solution used.

## Discussion

Our findings show that it is challenging to reconstruct reliable estimates of FC with low-density EEG. The low density setup leads to a considerable localization error in the reconstructed FC when source localization is performed. Furthermore, analyzing sensor FC without unmixing is unable to track FC correlates of motor performance. Yet, when high quality recordings are available and source FC is computed with MVBF, correlations with motor performance remained present even when using template head models and 19 electrodes, such suggesting that low-density montages may suffice in some cases for tracking FC as a neural correlate of behavior performance.

In accordance with the literature (Sekihara et al. 2005; Hadjipapas et al. 2005; Guggisberg et al. 2011), we observed superior localization accuracy of MVBF compared to sLORETA in both simulations (Fig. 2A) and real data (Fig. 3A, B). This can be explained with the fact that it adapts to the data and with its superior handling of noise, leading to more precise and focal reconstructions (Sekihara et al. 2005; Hadjipapas et al. 2005). In the case of FC calculations, a lack of focality leads to a greater susceptibility of inducing “ghost interactions”, i.e., spurious interactions reconstructed in the vicinity of true interactions due to signal mixing which is incompletely removed by the inverse solutions (Palva et al. 2018). Together, this leads to issues when reconstructing FC with non-adaptive inverse solutions such as sLORETA.

It is, however, important to note that the reconstructions obtained with low-density sLORETA remain more similar to high-resolution MVBF than when using sensor-space FC without source localization (Fig. 3C).

The advantage of MVBF over other approaches was reduced when using low-density montages (Figs. 2 and 3). This is because the noise handling of MVBF depends on a sufficiently large number of sensors. Thus, the localization error of MVBF is proportional to the sensor coverage (Steinsträter et al. 2010). Furthermore MVBF depends on head model accuracy. If the head model is not precise enough, the real source is simply considered as noise and ignored (Van Veen et al. 1997; Steinsträter et al. 2010). Conversely, sLORETA is a non-adaptive filter where the weights do not depend on the data. Hence, sLORETA’s location accuracy is less electrode-density-dependent. Yet, reducing the number of sensors reduces the number of available data points for reconstructing the source activity, leading to a redundancy of the estimated FC among the source regions even when using sLORETA.

Our simulations showed that MVBF led to lower correlations between the simulated coupling strength and the reconstructed FC magnitude than sLORETA, at least in case of high density montages (Fig. 2B). MVBF has known difficulties in reconstructing the time course of the source signal (Huang et al. 2014). Beamformers presume that the reconstructed sources are uncorrelated in time. Source activities are seen as orthogonal to one another in the time domain making source interdependencies non-existent (Van Veen et al. 1997; Sekihara et al. 2002; Hadjipapas et al. 2005). Previous studies have demonstrated that beamformers only show acceptable accuracy in reconstructing correlated sources when the correlation was transient, lasting less than 30–40% of the analysis duration. When exceeding 40%, temporal bias and signal cancellation appears (Hadjipapas et al. 2005). As the signals in our simulations did show some correlation, this may have introduced distortions of the reconstructed time series, and thus led to a reduced ability to quantify coupling strength. This is probably also the case in real recordings where some areas may show longer lasting temporal correlations.

Despite the disadvantages of MVBF, we observe that even when using template head models and low density montages, source FC reconstructed with MVBF gave best overall ability to capture FC correlates of motor performance (Fig. 4). In particular, FC reconstructions obtained in source space with MVBF were clearly superior in finding correlates of motor performance than sensor FC or FC obtained after ICA. Unlike sensor FC or ICA, MVBF uses information about head geometry to unmix the underlying neural signals, which gives it a precious advantage for applications on diagnosis and treatment of FC changes.

Yet, we have to bear in mind that we were unable to find such correlates of motor performance in a second, independent dataset which was recorded with a low-density EEG system using more convenient dry-gel electrodes (Fig. 5). This is most likely due to more noisy recordings obtained with this system, which is evident already by the fact that 5 out of 20 subjects had to be excluded for this reason. The impedance between skin and electrodes is larger when using dry or dry-gel electrodes, which deteriorates signal quality and further complicates the already difficult task of reconstructing FC from low-density EEG. This also generally raises a caveat with regards to the robustness of FC reconstructions when using low-density montages.

Nevertheless, our results suggest that low-density EEG may in some instances be sufficient for applications for motor training to improve the participant’s performance. Both patients and healthy subjects in sport and music could benefit from neuromodulation of alpha-band FC, using neurofeedback. A low-density EEG then enables more comfort and ease-of-use for the end-user.

It is important to stress that our study was performed with the aim of potential clinical applications with a particular neural target. The assessment of other frequency bands, which often have lower SNR than the alpha band, and the usage of other indices of FC, may lead to different results. Furthermore, our study focused on FC correlates of motor performance. As FC correlates with performance also in other domains, it would be intersting to extend this to other functions. In these cases, the ability of low-density EEG to capture these correlates would need to be evaluated separately. Our analyses across all cortical ROIs suggests that many of the observations made for the motor cortex and many of the observed difficulties apply also to other brain aras.

## Conclusion

Our study illustrates the difficulties in reconstructing FC from low-density EEG. It is common practice to compute sensor FC among electrodes, and to attribute the resulting findings to certain brain areas. This study shows that the mixed signals recorded by each electrode precludes an estimation of FC at a given brain area. Furthermore, FC correlates of behavior which are robustly observed at the source level are lost when analyzing sensor FC. FC reconstruction from low-density EEG is more feasible when obtaining source localization with adaptive inverse solutions such as MVBF, although these reconstructions are less robust than the ones obtain from high-density EEG. These insights may enable new possibilities for training and learning in clinical practice and public usage.

## Data Availability

The datasets analyzed during the current study are not publicly available because the consent from the participants did not include public dissemination, but are available from the corresponding author upon reasonable request.

## Code Availability

Analyses have been done with open source Matlab toolboxes that can be downloaded freely as indicated in the manuscript.
